# Production and characterization of intracellular invertase from *Saccharomyces cerevisiae* (OL629078.1), using cassava-soybean as a cost-effective substrate

**DOI:** 10.1038/s41598-023-43502-2

**Published:** 2023-09-28

**Authors:** Oghenesivwe Osiebe, Isaac Olusanjo Adewale, Bridget Okiemute Omafuvbe

**Affiliations:** 1 Department of Biochemistry and Molecular Biology, Obafemi Awolowo University, Ile-Ife, Nigeria; 2 Department of Microbiology, Obafemi Awolowo University, Ile-Ife, Nigeria

**Keywords:** Biochemistry, Biotechnology

## Abstract

The growing global market for industrial enzymes has led to a constant search for efficient, cost-effective methods for their production. This study reports the production of invertase using inexpensive and readily available agro-materials. Starch-digesting enzymes extracted from malted unkilned sorghum were used to hydrolyze cassava starch supplemented with 2% whole soybean. The production of intracellular invertase by *Saccharomyces cerevisiae* OL629078.1 in cassava-soybean and yeast sucrose broth was compared. The purification and characterization of invertase produced using the low-cost medium were also reported. The results showed that there was a 4.1-fold increase in the units of invertase produced in cassava-soybean medium (318.605 U/mg) compared to yeast sucrose broth medium (77.6 U/mg). The invertase produced was purified by chromatographic methods up to 5.53-fold with a recovery of 62.6%. Estimation of the molecular weight with gel filtration indicated a molecular weight of 118 kDa. The enzyme demonstrated its maximum activity at 50 °C and there was no decrease in its activity following a 1-h incubation at this temperature. At a pH of 5.0, the enzyme demonstrated optimal activity and it maintained over 60% of its activity in the acid range (pH 3–6). The Michalis-Menten constants *K*_*m*_ and *V*_max_ of intracellular invertase were 5.85 ± 1.715 mM and 6.472 ± 2.099 U/mg, respectively. These results suggest that *Saccharomyces cerevisiae* grown on cassava-soybean is a viable, cost-effective alternative for commercial invertase production, which can be explored for biotechnological processes.

## Introduction

The catalytic nature of enzymes has made them invaluable for industrial applications, where speed, specificity, precise product formation, and high efficiency are paramount. The market for industrial enzymes was valued at 6 billion USD in 2017 and it's anticipated to reach 16.9 billion USD in 2027, growing at a compound annual growth rate of 6.8% between 2022 and 2027 (http://marketresearch.com/). Hydrolases including carbohydrase, protease, and lipases account for about 75% of the industrial enzyme market^1,2^. Carbohydrases comprising amylases, isomerase, and invertase, among others, are the largest group of enzymes in industrial applications between 2016 and 2022^3^ (http://marketresearch.com/). One of the largest consumers of commercial enzymes globally are food and beverage industries^4^.

Invertase (β-fructofuranosidase, E.C. 3.2.1.26) is a carbohydrase that catalyzes the hydrolysis of sucrose to yield glucose and fructose in equimolar proportion, known as invert sugar. It is one of the most frequently used food and beverage enzymes, owing to the numerous possible applications of its products^3,5^. The manufacture of non-crystalline sugar, known as high fructose syrup, used as a sweetener; the synthesis of short-chain fructooligosaccharides; and the manufacture of digestive aids in pharmaceutical industries, among other applications, have resulted in an ever-increasing global demand for the enzyme^5,6^. Consequently, there is a constant search for both efficient producers of invertase and improved cost-effective methods of enzyme production. Yeasts, especially *Saccharomyces cerevisiae*, *Aspergillus niger*, and *Pichia pastoris* have been the major biological machinery for invertase production through cellular anabolism^7^. Therefore, low-cost readily available biomass is constantly explored for invertase production. Among the options of carbohydrate polymers explored, the hydrolysis of cassava starch has been central industrially for obtaining fermentable sugars suitable for microbial enzyme production. Conventional methods for the hydrolysis of starch account for 30–40% of the cost associated with starch-based industrial processes^8^.

Cassava (*Manihot esculenta* Crantz) is a perennial crop that is widely cultivated around the globe and valued for its starch-rich roots, which serve as renewable starch stores. Cultivation of cassava for starch extraction on a commercial scale is a steadily growing trend around the globe, especially in Vietnam, Asia, Paraguay, Brazil, and the Americas^9^. Hydrolysis of cassava starch to obtain reducing units suitable for conversion to various value-added products such as biogas and ethanol has been reported^9,10^.

The steadily increasing demand for industrial enzymes requires a constant search for cost-effective, readily available, and efficient methods of enzyme production. Cassava, a renewable starch store that is readily available all year round globally, due to its ease of propagation, drought resistance, good adaptability properties, and high yield, is a potential biomass for industrial applications^11^. However, concerns such as the cost of starch hydrolysis and medium-induced differential protein expression hamper the use of cassava-based medium for enzyme production. Therefore, it is necessary to study the invertase production of a known hyperproducing strain in cassava medium and a standard medium for invertase production (yeast sucrose broth). This study aimed to prepare a low-cost, alternative substrate for invertase production and study the production of invertase in the medium. Purification and characterization of the enzyme produced are also reported.

## Materials and methods

### Isolation and identification of yeast

The yeast used in this study, *Saccharomyces cerevisiae* OL629078.1 was isolated and identified by phenotypic and genotypic procedures, as reported in a previous study^12^. The ribosomal Internal Transcribed Spacer (ITS) sequence of the isolate available at https://www.ncbi.nlm.nih.gov/nuccore/OL629078.1 was used for phylogenetic analysis. Phylogenetic analysis tree was done by aligning the ITS sequences using the online Cluster Omega platform (https://www.ebi.ac.uk/Tools/msa/clustalo/) and the tree format generated was exported and visualized on the Interactive Tree of Life (iTOL) online platform (https://itol.embl.de/).

### Hydrolytic enzyme extraction and media preparation

The authors had permission to collect the plants used in this study, and the use of plants in the present study complies with all necessary international, national, and institutional guidelines. Sorghum grains, soybeans, and fresh cassava tubers were purchased from a local market in Ile-Ife. Sorghum grains were steeped for 24 h in distilled water, with the water being changed every 6 h. The steeped grains were then transferred to a malting chamber at room temperature for 72 h, and moistened periodically. Starch-digesting enzyme was extracted from malted unkilned sorghum grains by mechanical homogenization in 50 mM phosphate buffer, pH 6.0, at a ratio of 3:7. The homogenate was then centrifuged at 10,000 × *g*, and the supernatant was collected. Amylolytic enzyme activity in the supernatant was assayed following a previously described method^13^. One unit of enzyme was defined as the amount of enzyme that liberated reducing sugar equivalent to 1µmole of glucose per minute from starch at room temperature. Fresh cassava tuber (30% w/v) and soybean (2% w/v) were washed, pulverized, and suspended in distilled water containing 1 mM calcium chloride to form a mash. The starch in the prepared mash was hydrolyzed using amylolytic enzyme extract (2.356 ± 1545 U/mg), at a concentration of 6 ml per 100 ml of mash (141 U/l). This concentration was based on preliminary experiments (data not shown). The extract was added to the mash to form a mash-enzyme mixture, which was then incubated for 1 h with occasional stirring at 50 °C for hydrolysis. This was followed by filtration to obtain cassava-soybean medium (LCSM). The amount of reducing sugar due to hydrolysis was determined by measuring the amount of reducing sugar present in the mash before and after hydrolysis, as well as the amount of reducing sugars present in the crude enzyme extract by the Nelson-Somogyi method^14^.

1 $${\text{Reducing sugar due to hydrolysis }} = X{-}Y$$

where *X* = reducing sugar present after hydrolysis, *Y* = sugar present before hydrolysis + reducing sugar contributed by crude enzyme extract

### Invertase production in prepared medium and yeast sucrose broth

The standard medium for invertase production, yeast sucrose broth (YSB), containing 2% sucrose, 0.1% potassium dihydrogen phosphate, 0.4% yeast extract, and 0.05% magnesium sulfate heptahydrate was prepared^15^. A loop full of 18–24 h old cells of *Saccharomyces cerevisiae* was inoculated into 10 ml of sterile LCSM and YSB media and incubated for 24 h at 28 °C to obtain starter cultures. Media for invertase production (LCSM and YSB) were inoculated with 1 × 10^6^ cells/ml from appropriate starter cultures and incubated at 28 °C for 72 h. After incubation, the cells were harvested by centrifugation at 10,000 × *g* for 30 min at 4 °C. The harvested cell pellets were mechanically homogenized with acid-washed sea sand in 0.02 M Tris–HCl buffer pH 7.5 (1:1:2 respectively), as outlined in the study by Alegre et al.^16^. Homogenate obtained was subjected to centrifugation at 4 °C for 30 min at 10,000 × *g* and the amount of invertase and the protein concentration in the resulting supernatant were assayed at 28 °C.

### Invertase assay and determination of protein concentration

Invertase activity was measured by estimating the amount of reducing sugar released when the enzyme was incubated with sucrose at 28 °C for 10 min using the Nelson-Somogyi method^14^. The reaction mixture consisted of 143 M sucrose, which was dissolved in 0.08 M sodium acetate buffer at pH 4.7^12^. The control experiment was carried out using boiled enzyme preparation. One unit of enzyme was defined as the amount of the enzyme that catalyzed the release of reducing sugar equivalent to one micromole of glucose per minute from sucrose (substrate). The amount of protein was measured by the Bradford method^17^ using bovine serum albumin as the standard protein.

### Invertase purification and molecular weight determination

The crude enzyme (2 ml) was loaded onto a 1 × 20 cm chromatographic column packed with CM Sephadex C-50 resin. The column had been equilibrated with 0.01 M sodium phosphate buffer, containing 5% glycerol at pH 7. Elution was performed at a flow rate of 10 ml/h using the same buffer system, and a linear gradient of sodium chloride (ranging from 0 to 1 M), was applied for elution of bound proteins. During the elution process, 1.0 ml fractions were collected, and protein elution and enzyme activity in each collected fraction was monitored. The fractions exhibiting high invertase activity were combined and subsequently subjected to further fractionation based on size. The pool obtained from ion exchange was layered on a Sephadex G-100 chromatographic column (1 × 40 cm) that was equilibrated with 0.01 M Tris–HCl buffer pH 7.0 and fractions of 1.0 ml each were collected at a flow rate of 11 ml/h. Elution of protein at 280 nm and invertase activity in each fraction collected was measured as earlier described. Fractions that had high invertase activity were combined and used for subsequent biochemical analysis. The molar weight of invertase was estimated using a Sephacryl *S*-300 chromatographic column (1 × 40 cm). Calibration of the column was carried out using chymotrypsinogen (25,000 Da), ovalbumin (45,000 Da), bovine serum albumin (67,000 Da), alkaline phosphatase (140,000 Da) and glucose oxidase (160,000 Da).

### Effects of temperature, thermal stability pH, and kinetic parameters of invertase

To study the impact of temperature, the enzyme was added to the reaction mixture and incubated over a range of temperatures (10–70 °C). The enzyme thermal stability was examined by incubating the enzyme at different varied temperatures (20–60 °C) for 1 h. Samples were taken at 10-min intervals and analyzed under predetermined conditions to ascertain the residual activity. The initial enzyme activity before incubation was regarded as 100% for the purpose of this experiment. To investigate the impact of pH on invertase activity, the enzyme was examined in different buffer systems at varying pH levels ranging from 3 to 10. The values of the kinetic parameters *K*_*m*_ and *V*_max_ were obtained through the use of non-linear regression (GraphPad prism 7) with sucrose concentrations ranging from 0 to 200 mM.

## Results

### Microbial identification

**Figure 1:**
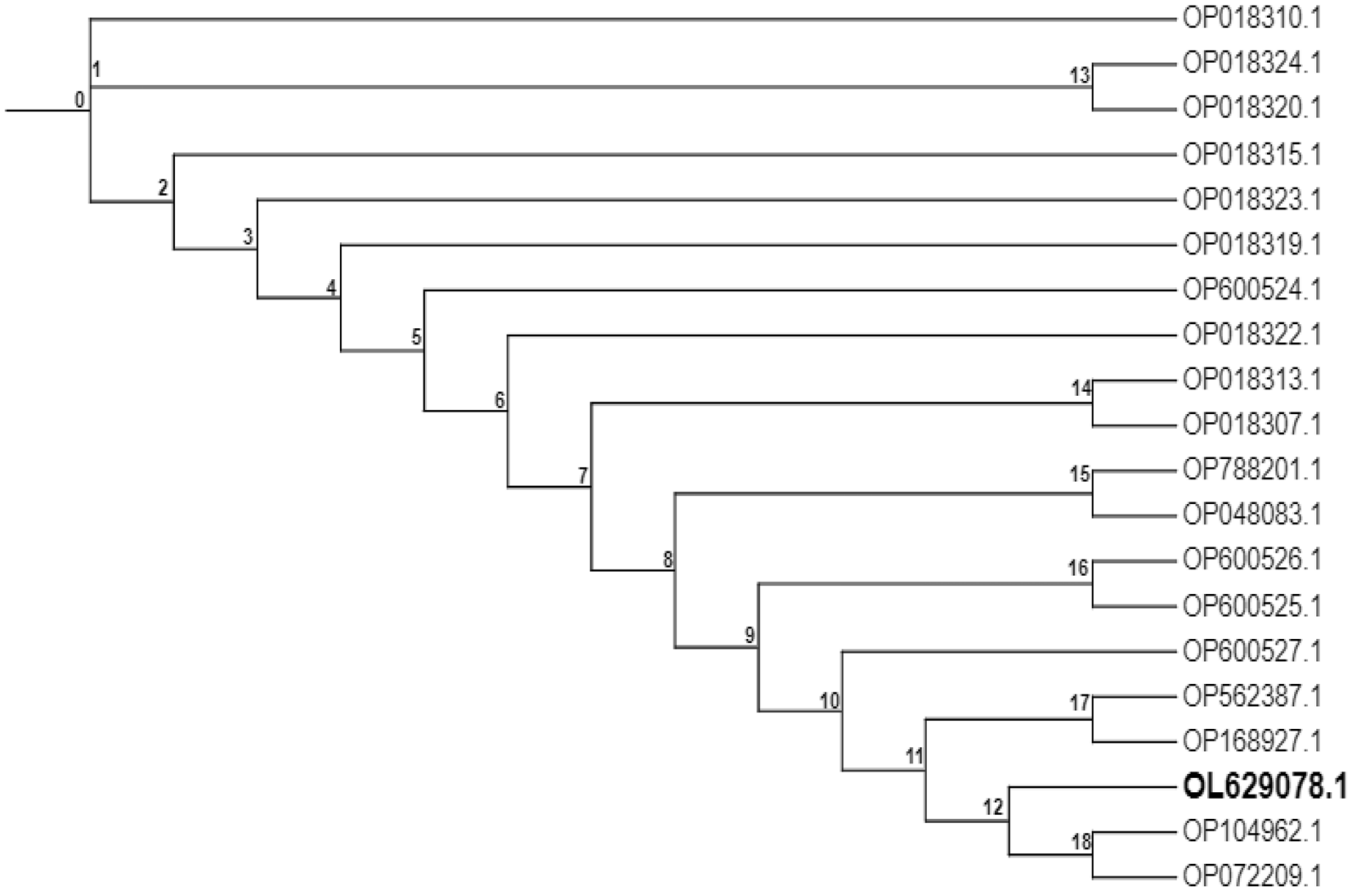
Phylogenetic tree of *Saccharomyces cerevisiae* OL629078.1 in relation to other strains. Isolate clustered with *Saccharomyces cerevisiae* OP104962.1 and *Saccharomyces cerevisiae* OP072209.1 at node 12, hereby having significant strain similarity. The isolate further diverges from other strains at node 12.

Phylogeny of the isolate is presented in Fig. 1. The isolate sequence OL629078.1 shared a similar node (node 12) with *Saccharomyces cerevisiae* OP104962.1 and *Saccharomyces cerevisiae* OP072209.1 and further diverged away from other strains.

### Amylolytic induction and mash hydrolysis

**Figure 2:**
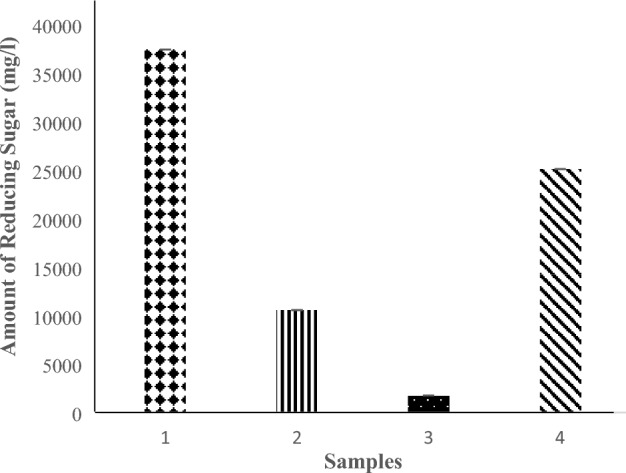
Amount of reducing sugar present in mash before hydrolysis, after hydrolysis, and the crude enzyme source. 1. Total amount of reducing sugar present in mash after hydrolysis. 2. Amount of reducing sugar present before hydrolysis. 3. Amount of reducing sugar present in malted sorghum. 4. Amount of reducing sugar due to hydrolysis. Values are the mean of three determinations; bars represent the standard error of mean.

Amylolytic enzyme specific activity in the crude extract of malted sorghum was 2.356 ± 1545 U/mg. The enzyme extract was able to hydrolyze cassava starch present in the mash, the reducing sugar content before and after hydrolysis and reducing sugar contributed by crude amylolytic enzyme extract are presented below in Fig. 2. Hydrolysis yielded 25.1 g/l of reducing sugar.

### Invertase production and purification

**Figure 3:**
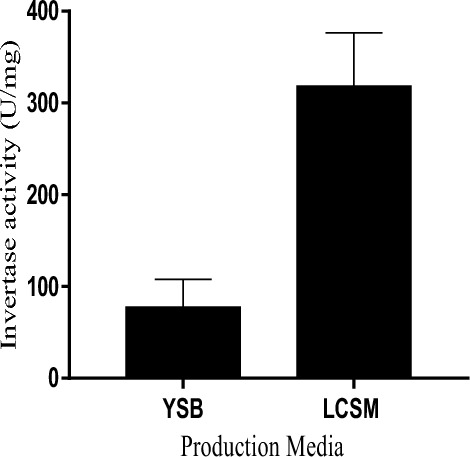
Effect of media on invertase production by *Saccharomyces cerevisiae* (OL629078.1). YSB: Yeast sucrose broth. LCSM: Liquefied cassava-soybean media. *Saccharomyces cerevisiae* was inoculated into the sterile media for 72 h at 28 °C, cells were then harvested and invertase produced was extracted and quantified as described above. Values are the mean of three determinations; bars represent the standard error of mean.

**Figure 4:**
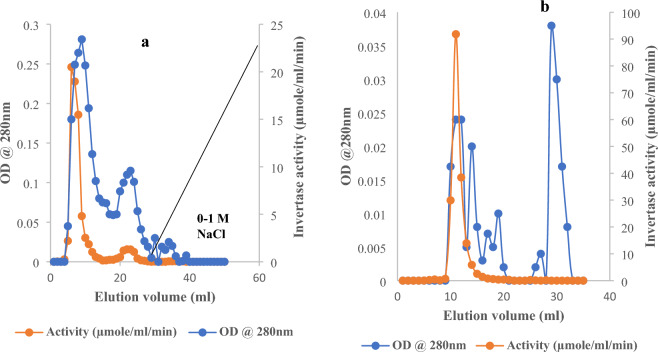
Elution Profile of intracellular invertase form *Saccharomyces cerevisiae* (OL629078.1). (**a**) Elution profile of invertase on CM-Sephadex C-50 ion-exchange chromatographic column. The column was equilibrated with 0.01 M sodium phosphate buffer pH 7.0, which also served as elution buffer, a linear gradient of sodium chloride was applied to elute bound proteins. (**b**) Elution profile of partially purified invertase on Sephadex G-100 chromatographic column. The gel filtration column was equilibrated with 0.01 M Tris–HCl buffer pH 7.0, the same buffer was used to load and elute the sample.

The production of invertase by *Saccharomyces cerevisiae* in YSB and LCSM is shown in Fig. 3. There was a 4.1-fold increase in the units of invertase produced by *Saccharomyces cerevisiae* in LCSM (318.605 U/mg) when compared to YSB (77.6 U/mg). Table 1 shows the invertase production in LCSM in comparison to production in glucose, sucrose, and other low-cost alternative carbon sources that have been explored for invertase production in the literature. *Saccharomyces cerevisiae* showed higher expression of intracellular invertase when cultured in LCSM. Therefore, invertase extracted from the isolate grown in this medium was purified and characterized. The Purification summary is presented in Table 2. Upon purifying crude invertase on CM-Sephadex C-50, the results revealed the presence of two peaks of invertase activities. Both peaks were not bound to the cationic exchanger, as indicated in Fig. 4a. However, further purification was not performed on the minor peak. Purification of the major peak on Sephadex G-100 (Fig. 4b) produced a single peak of invertase activity, with a recovery of 62.6% and an increase in purity by 5.53-fold. Native molecular weight estimation on gel filtration chromatography indicated a molecular weight of 118.3 kDa.

**Table 1:** Various carbon sources for microbial invertase production.

Carbon source	Units of enzyme produced (U/ml)	Organism	Reference
LCSM	64.4	*Saccharomyces cerevisiae* OL629078.1	This study
Sucrose	50	*Saccharomyces cerevisiae* CAT-1	Nascimento et al.^18^
Glucose	21	*Saccharomyces cerevisiae* CAT-1	Nascimento et al.^18^
Pineapple peel	24.2	*Aspergillus niger* IBK1	Oyedeji et al.^6^
Wheat bran	19.1	*Aspergillus caespitosus*	Alegre et al.^16^
Pineapple crown	9.7	*Aspergillus carbonarius* PC-4	Batista et al.^5^
Rice straw	4.2	*Aspergillus caespitosus*	Alegre et al.^16^
Sugarcane bagasse	2.2	*Aspergillus caespitosus*	Alegre et al.^16^
Orange peel	0.22	*Penicillium* spp*.*	Nehad and Atalla^19^
Beet peel	0.18	*Penicillium* spp*.*	Nehad and Atalla^19^
Apple peel	0.16	*Penicillium* spp*.*	Nehad and Atalla^19^

**Table 2:** Purification summary of *Saccharomyces cerevisiae* intracellular invertase.

Procedure	Volume (ml)	Activity (U/ml)	Total Activity (U)	Protein concentration (mg/ml)	Total Protein (mg)	Specific Activity (U/mg)	Purification Fold	Yield (%)	Purification fold
Crude enzyme	3.6	64.4	231.8	1.3	4.68	49.53	1	100	1
CM-Sephadex C-50	21.42	12.5	267.75	0.13	2.78	96.31	1.94	115.5	1.94
Sephadex G-100	33	4.4	145.2	0.016	0.53	273.96	5.53	62.6	5.53

### Effects of temperature, thermal stability, pH and on invertase activity and determination of kinetic parameters of purified invertase

**Figure 5:**
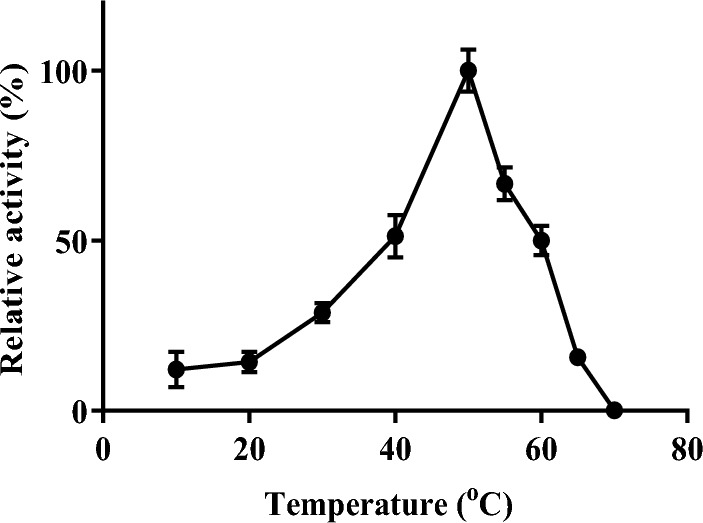
The effect of temperature on the activity of intracellular invertase extracted from *Saccharomyces cerevisiae* (OL629078.1). The enzyme was incubated with sucrose in assay buffer (0.08 M sodium acetate buffer, pH 4.7) at varied temperatures and the activity of invertase was estimated as described earlier. Values are the mean of three determinations; bars represent the standard error of mean.

**Figure 6:**
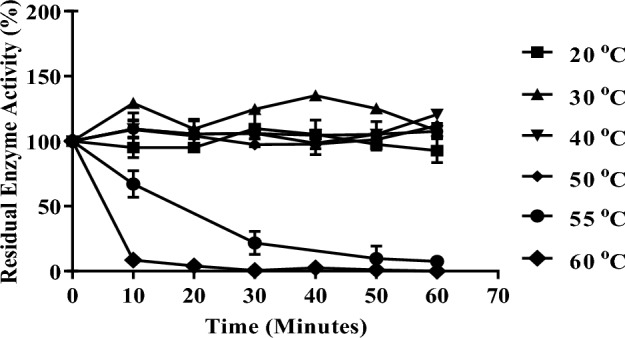
Effect of temperature on the stability of intracellular invertase from *Saccharomyces cerevisiae* (OL629078.1). Invertase was incubated without substrate at various temperatures ranging from 20 to 60 °C for 1 h. Samples were drawn at 10 min intervals and the residual enzyme activity was determined by assay as described earlier. The initial enzyme activity before incubation was regarded as 100%. Values are the mean of three determinations; bars represent the standard error of mean.

**Figure 7:**
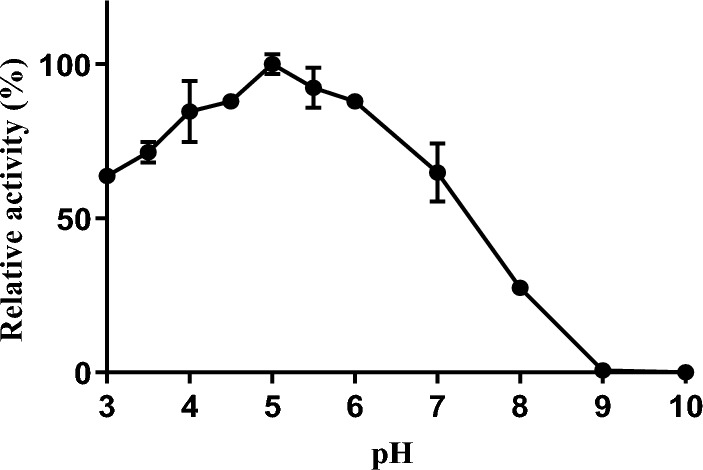
The effect of pH on activity of intracellular invertase from *Saccharomyces cerevisiae* (OL629078.1). Purified invertase was incubated with sucrose at different pH (3–10) and the enzyme activity was assayed at 28 °C as earlier described. Values are the mean of three determinations; bars represent the standard error of mean.

**Figure 8:**
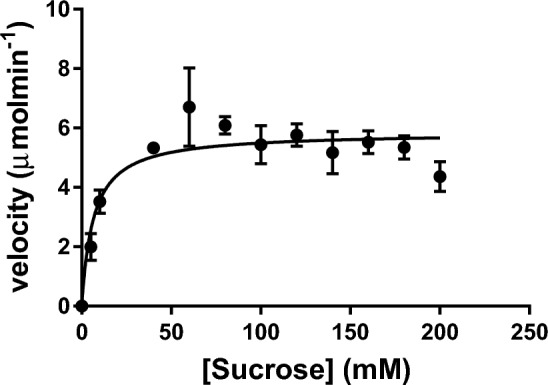
The non-linear regression curve for determination of kinetic parameters of invertase extracted from *Saccharomyces cerevisiae* (OL629078.1). Sucrose concentration was varied between 5 and 200 mM keeping all other assay conditions as predefined. Values are the mean of three determinations; bars represent the standard error of mean.

The result of the temperature effect on invertase activity is presented in Fig. 5. The enzyme showed the highest activity at a temperature of 50 °C. The thermal stability study on invertase is shown in Fig. 6. Invertase was stable between 20 and 50 °C and there was no loss of activity when the enzyme was incubated for 60 min at these temperatures. Invertase lost 33% of its initial activity after an incubation period of 10 min at 55 °C, at 30 min of incubation the enzyme lost 79% of its activity, and complete inactivation was observed after 50 min of incubation. At 20 min of incubation at 60 °C invertase was observed to be completely inactive and no residual activity was detected on further incubation at this temperature. The enzyme showed good activity in the acidic range (Fig. 7) and an optimum pH of 5.0. The kinetic parameters; *K*_*m*_ and *V*_max_ of intracellular invertase were 5.85 ± 1.715 mM and 6.472 ± 2.099 U/mg respectively (Fig. 8).

## Discussion

Palm wine hosts a diverse community of microorganisms, and various studies have isolated microorganisms with potential commercial applications such as bioethanol and enzyme production from its microbial pool^20^. The phylogenetic tree of the palm wine yeast isolate used for enzyme production in this study is shown in Fig. 1. The isolate shares a similar node with *Saccharomyces cerevisiae* OP104962.1 and *Saccharomyces cerevisiae* OP072209.1 and further diverges away from other strains. It shares a close evolutionary relationship with *Saccharomyces cerevisiae* OP104962.1 and *Saccharomyces cerevisiae* OP072209.1. *Saccharomyces cerevisiae* OL629078.1, which was explored here for enzyme production, has been previously shown to be a natural invertase hyperproducing strain^12^.

Microbial enzyme production relies on the conversion of simple and fermentable sugars into proteins by the anabolic process of cells^21^. Equation 1 above shows that the amount of reducing sugar due to hydrolysis is equivalent to 25.1 g/liter. This confirms that amylolytic enzymes from malted unkilned sorghum are effective for the hydrolysis of cassava starch. Luz et al.^22^ used α-amylase extracted from malted barley and millet to hydrolyze cassava starch, and the concentration of reducing sugar they reported was lower than the amount obtained in this study. Since starch hydrolysis accounts for about 40% of the total cost incurred for starch-based products, this method of hydrolysis can significantly reduce production costs and facilitate the industrial utilization of cassava^8,23^. The extraction pH and hydrolysis temperature used in this study are the optimal conditions for α-amylase. However, glucoamylase is not completely inactivated at these conditions and also contributes to hydrolysis. Therefore, the possible sugars present in the mash are mainly oligosaccharides and α-limit dextrins such as maltose and maltotriose, with some glucose^22,24^. There was a 4.1-fold increase in units of invertase produced by *Saccharomyces cerevisiae* in LCSM when compared to YSB. This indicates that LCSM is better suited for invertase production than YSB. In comparison to carbon sources such as glucose, sucrose, and various low-cost alternative carbon sources that have been explored for invertase production (Table 1), LCSM is more suitable for the enzyme production. In addition to being a suitable substrate for invertase production, the results also suggest that cassava-soybean medium may contain intracellular invertase inducers, which would explain the medium-induced increase in enzyme expression. Changes in microbial protein expression due to media composition have been previously observed in the literature^25^. Low-cost readily available feedstock is in constant demand for commercial enzyme production, hence LCSM can be explored for subsequent biotechnological applications.

The enzyme was unbound to the cation exchanger and eluted as a single peak of activity on a Sephadex G-100 column (Fig. 4a–b). These findings are consistent with previous reports. Rashad et al.^26^ reported that invertase was bound by an anion exchanger and gave a single peak of activity on a Sephacryl *S*-300 column. Purification reached a fold of 5.3 with a recovery yield of 62.6%. The purification fold is consistent with previously reported data, but the recovery yield reported here is significantly higher and is therefore suitable for large-scale production^6,26,27^. The molecular weight of the enzyme is consistent with the value previously reported for invertase from other microbial sources. Invertase from *Penicillium expansum* was reported to have a molecular weight of 110 kDa by Kashif et al.^27^ and a similar value was also reported for invertase from *Saccharomyces cerevisae* EMS-42 by Aslam and Ali^28^.

The optimum temperature for the enzyme was 50 °C. There was no loss in enzyme activity when it was incubated without substrate at this temperature for 1 h, and it was completely inactivated at 60 °C. This indicates the suitability of the enzyme for biotechnological applications. Bhalla et al.^29^ reported an optimum temperature of 40 °C, stability between 30 and 50 °C, and complete inactivation at 70 °C for invertase from *Saccharomyces cerevisiae* SAA-612. Avila et al.^30^ reported an optimum temperature of 50 °C and a decline in stability at higher temperatures when *Candida guilliermondii* invertase was characterized. Oyedeji et al.^6^ reported maximal activity at 60 °C for invertase from *Aspergillus niger* IBK1, while Shankar et al.^31^ reported an optimum temperature of 30 °C for the enzyme from *Saccharomyces cerevisiae* MTCC 170. The range of optimum activity temperatures for invertase varies widely in the literature. However, thermostable enzymes are generally preferred for industrial applications^32^. Therefore, the invertase reported here is a potential industrial enzyme, considering its optimum temperature and thermal stability.

The optimum pH for *Saccharomyces cerevisiae* invertase in this study (5.0), is consistent with previous studies. Avila et al.^30^ reported an optimum pH of 5.0 when characterizing invertase from *Candida guilliermondii*. Nehad and Atalla^19^ reported an optimum pH of 6.0 for immobilized invertase. Barbosa et al.^33^ reported optimal pH of 4.0 and 4.5 for *Rhodotorula mucilaginosa* and *Saccharomyces cerevisiae* invertases respectively. Although Zhou et al.^34^ reported the isolation of alkaline invertase with an optimum pH of 8.0 from *Bacillus* sp. HJ14, microbial invertases are predominantly acid invertases. *Saccharomyces cerevisiae* OL629078.1 invertase had optimum activity in the acid range (pH 3–6), mainlining over 60% of its activity in this range. This is advantageous in industrial applications such as invert syrup manufacture^6^.

Michalis-Menten constants *K*_*m*_ and *V*_max_ of invertase was 5.85 ± 1.715 mM and 6.472 ± 2.099 U/mg respectively, indicating that the enzyme has strong affinity for sucrose. Dal Maso et al.^35^ characterized the invertase of a filamentous fungi and recorded *K*_*m*_ and *V*_max_ of 3.91 mM and 20.24 µmol/min.ml respectively. Bhalla et al.^29^ reported *K*_*m*_ and *V*_max_ values of 11 mM and 434.7 U/mg respectively for invertase from *Saccharomyces cerevisiae* SAA-612. Oyedeji et al.^6^ reported *K*_*m*_ and *V*_max_ of 21.93 mM and 35.71 U/min/ml respectively for invertase from *Aspergillus niger* IBK1. Comparing the values with those of other microbial invertases cited in the literature, the *K*_*m*_ value recorded for *Saccharomyces cerevisiae* invertase is within the recorded range, however, the *V*_max_ value is not similar. This difference could be due to different enzyme sources or variations in experimental conditions.

## Conclusion

In conclusion, *Saccharomyces cerevisiae* OL629078.1 is a promising strain for high-titer invertase production using liquefied cassava-soybean medium, a low-cost, easily available, and renewable substrate. The enzyme produced has suitable properties for industrial utilization owing to its thermostability and acidic optimal pH. Therefore, high catalytically efficient invertase, suitable for industrial applications, could be obtained in commercial quantities from *Saccharomyces cerevisiae* cultured in liquefied cassava-soybean medium.

## Data Availability

Sequence data can be accessed at GenBank National Centre for Biotechnology Information database [https://www.ncbi.nlm.nih.gov/nuccore/OL629078.1].
